# Do young people perceive their smartphone addiction as problematic? A study in Danish university college students

**DOI:** 10.1016/j.heliyon.2023.e20368

**Published:** 2023-09-25

**Authors:** Gitte Frydenlund, Julie Dalgaard Guldager, Katrine Oertel Frederiksen, Heidi Klakk Egebæk

**Affiliations:** a Department of Physiotherapy Education, University College South Denmark, Degnevej 16, 6705 Esbjerg, Denmark; b Research Department, University College South Denmark, Haderslev, Denmark; c Unit for Health Promotion Research, Department of Public Health, University of Southern Denmark, Esbjerg, Denmark; d Centre for Research in Childhood Health, Institute of Sports Science and Clinical Biomechanics, University of Southern Denmark, Odense, Denmark; e Centre for Clinical Research and Prevention, Section for Health Promotion and Prevention, Bispebjerg and Frederiksberg Hospital, Nordre Fasanvej 57, 2000 Frederiksberg, Denmark

**Keywords:** Smartphone addiction, Mental health, Physical health, Perceived health, Students, Behavior change

## Abstract

As smartphone addiction has been linked to poor mental health and lower levels of physical activity, it is of public health interest to explore the behavior behind problematic smartphone use and develop interventions to reduce smartphone use. This study aimed to investigate the risk of smartphone addiction and examine perceived problematic smartphone behavior among university college students.

This online survey conducted amongst 1251 Danish University College students studied smartphone addiction in conjunction with physical- and mental health dimensions. The risk of smartphone addiction was estimated using the *Smartphone Addiction Scale—Short Version (SAS-SV*). The main results are presented as odds ratios from multivariate logistic regressions.

One in four (23%) were at high risk of smartphone addiction. Of this high-risk group, 74% identified their smartphone behavior as problematic, with 91% having considered reducing their smartphone use.

Students with a high risk of smartphone addiction perceiving their behavior as problematic were more likely to report low mental health and well-being.

In conclusion, students at high risk of smartphone addiction acknowledge their problematic behavior and have actively considered behavior modifications. This knowledge can enable teachers, parents, and social and health workers to understand that a majority of heavy smartphone users are open to reducing their smartphone usage, albeit with the appropriate support.

## Highlights

• Few studies have assessed whether young people with a high risk of smartphone addiction perceive their behavior as problematic
• One in four students reports a high risk of smartphone addiction
• The majority of these perceive their smartphone behavior as problematic and consider reducing their smartphone use
• Students with high levels of smartphone use perceiving their behavior as problematic, report low mental health and well-being

## 1 Introduction

The widespread use of smartphones have increased significantly since the first iPhone was launched to the public in 2007 [1]. Smartphones have become ubiquitous, making us potentially online, connected, and available anytime and everywhere [2]. In Denmark, for instance, 90% of the population owns a smartphone, and most children obtaining their own by age 9 [3]. This early exposure to screen media use is higher than in countries like the U.S. and the U.K., with 42% of U.S [4]. and 50% of British [5] 10-year-old children owning a smartphone, compared to Denmark's 93% [6]. Research has shown that early acquisition of smartphone among undergraduate University students correlates with subsequent smartphone addiction [7]. Cross-sectional studies have also linked excessive screen time, including smartphones, with multiple aspects of poor mental health in young people, like depression [[8], [9], [10], [11], [12]], stress [[12], [13], [14]], anxiety [9], low self-esteem [15], and loneliness [12,16]. However, excessive smartphone use can also reduce loneliness and relieve stress for people using online platforms to communicate their feelings, anxieties, and problems [11]. Additionally, cross-sectional studies have found associations between smartphone addiction and decreased physical activity [17,18], increased sedentary behavior, and decreased participation in regular sports [19]. That said, not all smartphone usage is problematic [20], but -excessive and compulsive usage might lead to problematic smartphone use [21]. This has previously been referred to as “smartphone addiction” or “problematic smartphone use” [22]. Smartphone addiction has been studied based on previous internet addiction research [23]. Smartphone use is distinguished from traditional internet use on computers or laptops because smartphones allow users to access the internet continuously regardless of time and space [24]. Smartphone addiction is characterized by features such as daily-life disturbance, positive anticipation, withdrawal, cyberspace-oriented relationship, overuse, and tolerance [25].

Research into problematic internet use or smartphone use has predominantly focused on symptoms and risk factors [9,14,16,26,27]. However, in a systematic review of scales to assess problematic smartphone use by Harris et al. [28], it is -suggested that future research should delve deeper into the behavior that constitute problematic smartphone use. Understanding the behavior behind problematic smartphone use is crucial for users, professionals, and health authorities in planning prevention and treatment.

“The Stages of change” model by Prochaska and DiClemente [29] is a recognized and often-used theory of behavioral change in health promotion. According to the model, acknowledgement of the problematic health behavior is one of the most important factors separating “the contemplation stage” from the “pre-contemplation stage” and thus is a crucial link for change to occur. Therefore, in interventions to reduce smartphone addiction, it would be essential to know if the target group perceives their behavior as problematic or not, because a non-recognition of problematic health behavior will be a major obstacle to potential behavior change [29].

Therefore, it would be particularly useful to characterize the group at high risk of smartphone addiction who perceive their behavior as problematic since this group might be more susceptible to possible interventions to reduce smartphone use.

Based on this, we aimed to answer the following research questions: 1) How common is a high risk of smartphone addiction among Danish university college students and, what characterizes this group? 2) To what degree do those identified as being at high risk of smartphone addiction perceive their behavior as being problematic? 3) Can mental and physical health differences be identified in students with a high risk of smartphone addiction who perceive their behavior as problematic?

## 2 Methods

### 2.1 Study design

This study was a quantitative cross-sectional study based on data from an online questionnaire for Danish university college students.

### 2.2 Participants

A convenient sample of university college students aged ≥18 years from 16 different study programs at University College South Denmark was recruited for this study.

### 2.3 Data collection

All persons listed as a University College South Denmark student in April 2022 were considered eligible (*n* = 6067). After removing duplicates (n = 142) and invalid email addresses (n = 225), a total of 5700 students were contacted by email and e-learning platforms (Intranet/E-learn) with an explanation of the study aim and circumstances for participation and a link to the online survey. A reminder was sent four, seven, and twelve days after the initial contact.

Data on gender, age, and study program distribution of the eligible study population (all active students at the University College South Denmark) was extracted from the University College South Denmark administration in order to compare the responders (the actual study population) to the background study population on these characteristics.

### 2.4 Content of the questionnaire

The questionnaire consisted of questions from existing validated questionnaires and items developed by the research team. The questionnaire was pilot tested for feasibility and face validity in a Delphi process by pre-testing on 34 students from 10 different study programs and through two focus group interviews (*n* = 10) as recommended by Vet et al. [30]. As a result, the survey underwent minor changes (e.g. to ensure all students could feel anonymous when answering the survey, a question about study year was removed, and age was transformed from a numerical to a categorical question).

Other studies have associated the selected variables with smartphone addiction [9,[17], [18], [19],[31], [32], [33], [34], [35], [36], [37], [38], [39]]. To our knowledge, no tool or questionnaire has been developed to catch the perception of whether or not individuals perceive their smartphone use as problematic and if they ever considered changing their behavior and reduce their smartphone use. This acknowledgement of problematic behavior and considerations of change is, according to the “Stages of Change theory”, crucial links for change to occur [29]. Based on the statements in the Theory (Stages of Change) and the clinical and empirical experience in the research group, we constructed the variables “Self-assessment of perceived problematic smartphone use” and “Considerations for reducing smartphone use”. Face validity and feasibility were tested in a pilot study mentioned above (not published).

The applied questions can be seen in Appendix 1 (Applied questions_English version) and Appendix 2 (Applied questions_Danish version). The questionnaire covered the following areas:

**Sociodemographic variables**: Students were asked about their *gender* (man, woman, or other) and *age* (<20, 20–24, 25–29, 30–34, 35–39, ≥40). Further, students were asked which *education/study program they currently follow*: 1) schoolteacher, 2) kindergarten teacher, 3) pedagogical assistant, 4) nurse, 5) midwife, 6) medical laboratory technologist, 7) laboratory technician, 8) occupational therapist, 9) physiotherapist, 10) dietitian, 11) social worker, 12) tax and public administration, 13) graphic communication, 14) English and digital marketing communication, 15) sound design, and 16) health administrative coordinator). For statistical analyses (Table 1, Table 3), age was categorized into ≤24, 25–34, and ≥35, and study programs 1–3 were categorized as education, 4–10 as health education, and 11–16 as society and administration.

Students were asked *how many people they live with* (0, 1, 2, 3, 4, 5, 6, ≥7), which was categorized as “none”, 1 person, or ≥2 persons for the analysis, and *how often they are together/out with friends outside of their study time* (0, 1, 2, 3, 4, 5, 6 or 7 days/week) which was categorized as 0–1 times/week, 2–3 times/week or >3 times/week for statistical analysis. Further, students were asked if *they have a student job* (yes/no), and *how many hours they work* per *week*.

**Smartphone ownership:** Students were asked if they *own a smartphone* (yes/no). Those who answered no were exempt from subsequent smartphone-related questions.

**Smartphone addiction:** Risk of smartphone addiction was assessed using the 10-item *Smartphone Addiction Scale—Short Version (SAS-SV)* [23], which encompasses five aspects: Daily-life disturbance, withdrawal, cyberspace-oriented relationship, overuse, and tolerance. Answers were reported on a 6-point Likert scale (from “strongly disagree” to “strongly agree”). The scale runs from 10 (low risk) to 60 (high risk), with thresholds for high risk of smartphone addiction set at ≥31 in men and ≥33 in women - as suggested by Kwon [23]. The SAS-SV scale has been demonstrated to be valid and applicable for the same age group as in our sample [40], it is translated into a Danish, and has been used in a Danish study on adults [41]. Sensitivity and specificity were originally examined by Kwon et al. [23], who found a sensitivity of 0.87 for boys and 0.88 for girls, and a specificity of 0.89 for both genders.

**Perceived problematic smartphone use:** Students' self-assessment of potentially problematic smartphone use were assessed with the question, *“To which degree do you agree with the following statement? I consider my use of my smartphone as problematic”.* Answers were reported on a 6-point Likert scale (from “strongly disagree” to “strongly agree”) to establish if students perceive their behavior as problematic. For analysis, the responses were dichotomized into “agree” (“slightly agree”, “agree” or “strongly agree”) and “disagree” (“strongly disagree”, “disagree” or “slightly disagree”).

**Smartphone reduction consideration**: Students were asked, “*How often do you consider reducing your smartphone use?*” with the response options “never or almost never”, “sometimes”, “most of the time”, or “all the time or almost all the time”. For analysis, due to uneven distribution, these four categories were collapsed into three categories as “never” (“never or almost never”), “sometimes”, or “often” (“most of the time”/”all the time or almost all the time”) as the last category “all the time or almost all the time” had very few representations (3.2%)

**Quantity of smartphone use**: To assess smartphone use, we used an item from a validated questionnaire on screen time, the SCREENS-Q [42]. Smartphone usage was estimated by analyzing how much time students spent on varied activities on their smartphones over a period of four weeks. We asked the students *how much time* (0 min, 1–29 min, 30–59 min, 1–2 h, 3–4 h, ≥5 h) during an average weekday and weekend day, respectively, they have spent during the last four weeks on six different types of activities on their smartphone (“watching TV”, “playing games”, “talking on the phone”, “social media”, “surfing the web”, and “other”). The detailed way of asking what they spent time doing on the smartphone is meant to support their memory and not to differentiate or categorize smartphone use. Data were transformed into a continuous variable by substituting each answer category with the median of each category (e.g. “1–29 min” = 15). The highest category (≥5 h) had no upper limit, but it was assumed to have the same minute range as the preceding category [6]. This transformation allowed calculation of total smartphone use in hours and minutes/day for an average weekday and weekend day, respectively, and a total time in hours and minutes/week (hours and minutes on a typical weekday multiplied by five, plus hours and minutes on a typical weekend day multiplied by two).

**Physical activity:** Students were asked *how often they participate in organized sport during a week*, and *how often they are physically active besides organized sport*, respectively (0, 1, 2, 3, 4, 5, 6 or ≥7 times/week). For statistical analysis, both variables were categorized as 0–1 times/week, 2–3 times/week, or >3 times/week.

**Health-related quality of life:** This was assessed through a question from the 36 Item Short Form Health Survey Questionnaire (SF36) [43]. “*In general, would you say your health is:”.* The answer was rated on a 5-point Likert scale (from poor to excellent) and categorized as poor (fair/poor) or good (excellent/very good/good) as suggested by others [44] for statistical analysis.

**Mental health and well-being:** The students' mental health and well-being were evaluated using the 5-Item World Health Organization Well-Being Index (*WHO5*). WHO5 is one of the most widely used tools for measuring self-perceived well-being on a global scale, and a previous systematic review has found adequate validity for all 18 included studies (sensitivity = 0.86, specificity = 0.81) [45]. Items were rated on a 6-point Likert scale (from “at no time” to “all the time”). The sum was multiplied by 4 to give the final score from 0, representing the worst imaginable well-being, to 100, representing the best imaginable well-being. The resulting scores were categorized into low well-being (score 0–50) and normal well-being (score 51–100) for statistical analysis according to the WHO-5 cut-off score, which has been recommended when screening for clinical depression [45].

**Self-esteem:** Participants' self-esteem was assessed using the 10-item Rosenberg Self-Esteem Scale (*RSES*). Previous studies has confirmed the validity and internal consistency of RSES [[46], [47], [48]]. Items were rated on a 4-point Likert Scale (from “strongly disagree” to “strongly agree”). The scores were summed with scores ranging from 0 to 30. The scores were categorized as low self-esteem (scores ≤15) and normal self-esteem (scores 16–30), as recommended by others [49] for statistical analysis.

### 2.5 Statistical analysis

Absolute and relative frequencies were used to describe the characteristics of the participating students (Table 1, Table 2). Pearson's Chi-Square was used to establish if the differences between groups of students of high vs. low risk of smartphone addiction were significant (Table 1, Table 2). The variance inflation factor was used to test for multicollinearity of the variables “Health-related quality of life”, “mental health and well-being*”,* and *“*self-esteem”. All assumptions of non-collinearity were met. Multivariate logistic regression analysis was applied to determine variables associated with perceived problematic behavior in high-risk students. Results were presented as odds ratios (OR) and 95% confidence intervals, and analyses were conducted separately for each independent variable (model 1) and concurrently (model 2) (Table 3). A supplementary multivariate analysis was performed using continuous variables where possible. Otherwise, the original categorization was used. This was done to check if the multiple logistic analysis lost statistical strength due to the categorization of the variables. The supplementary multivariate analysis did not contribute to any new results. Therefore, we chose to present the results from the multivariate logistic analysis with dichotomized variables, since these results are easier to interpret. IBM-SPSS for Windows version 28 was used to conduct the statistical analysis.

### 2.6 Ethics

This study was conducted in accordance with the Declaration of Helsinki. All respondents were assured that their participation was voluntary and that their responses would be anonymized. They were also informed that answering the questionnaire was considered informed consent and guaranteed that personal data legislation was followed. Data processing in the current project was regulated in the Danish Act on Research Ethics Review of Health Research Projects section 14, subsection 2 [50], which states that health research based solely on questionnaire surveys and register data is exempt from the obligation to notify the ethics committees. Ethical approval was therefore not obtained.

## 3 Results

### 3.1 Description of the sample

Overall, 1251 students fully (response rate 21.9%) and 1612 partly completed the questionnaire (response rate 28.3%). Participants were compared to the general study population of all active University College South Denmark students regarding gender, age, and study program. There was no significant difference between our sample and the general study population regarding study program. The gender distribution was significantly different (p > 0.01), with 81.6% women in our sample versus 77.4% women in the general study population. Our study population had a higher proportion of students aged ≤24 years (48.8%) and students aged ≥35 years (19.1%), and a lower proportion of students aged 25–34 (32.1%) compared to the general study population (45.1%, 14.0%, and 40.9% respectively) (p > 0.001).

The level of smartphone addiction among all the participants was 23.1% (n = 287). High risk of smartphone addiction was found in 20.6% of men and 23.6% of women. Further, the score of the SAS-SV ranged from 10 to 45 points with a mean score of 23 for men, and from 10 to 60 with a mean score of 27 for women.

The characteristics of the participating students are presented in Table 1. In our study population, most students were women (81%), and half (49%) were aged 24 or younger. Nearly three-quarters (72%) were living with others, and half (50%) spent time with friends besides their studies ≥2 days per week (Table 1). Half (47%) of the students attended a study program in education, and the rest in health education (34%) and society and administration (19%). A little over half (60%) had a student job and worked an average of 14 h per week (SD ± 8.8) (not shown in the table).

**Table 1:** Characteristics of participating students stratified according to risk of smartphone addiction.

		All		High risk of smartphone addiction		Low risk of Smartphone addiction		P-value
		*n*	%	*n*	%	*n*	%	
Overall *n*		1247–1612^a^	–	284–287^a^		952–956^a^		
**Gender**								0.326
Men		230	18.4	47	16.4	181	18.9	
Women		1018	81.4	240	83.6	775	81.1	
Other		<5	0.2	–	–	–	–	
**Age**								**0.028**
≤24 years		610	48.8	153	53.3	455	47.6	
25–34 years		402	32.1	95	33.1	305	31.9	
≥35 years		239	19.1	39	13.6	196	20.5	**
**Living with others**								0.480
None		345	27.6	74	25.9	269	28.1	
1 person		445	35.6	110	38.5	331	34.6	
≥2 persons		459	36.7	102	35.7	356	37.2	
Mean ±(SD) (no. of cohabitants)		1.5 (±1.4)	–	1.4 (±1.3)	–	1.5 (±1.4)	–	
**Together with friends besides study**								**0.010**
0–1 day/week		652	50.1	126	43.9	503	52.6	**
2–3 days/week		505	38.8	119	41.5	361	37.8	
>3 days/week		145	11.1	42	14.6	92	9.6	*
Mean ±(SD)		1.7 (±1.5)	–	1.9 (±1.5)	–	1.6 (±1.4)	–	
**Student job**								0.976
Yes		779	59.9	172	59.9	572	59.8	
**Smartphone ownership**								–
Yes		1602	99.4	287	100.0	956	100.0	
**Perceive smartphone behavior as problematic**								**<0.001**
Agree		538	35.3	213	74.2	225	23.5	
**Consider reducing smartphone use**								**<0.001**
Often		372	23.6	116	40.4	180	18.8	***
Sometimes		820	52.6	144	50.2	506	52.9	
No		372	23.8	27	9.4	270	28.2	***

*: <0.05, **: <0.01 ***: <0.001.

^b^ = *behind the percentages indicates significant differences in rates between the respective sub-group and both other groups.

a Sample size for the different analyses diverges due to varying numbers of valid answers for the change assessments.

Almost everyone (99%) owned a smartphone, nearly two-thirds (65%) did not perceive their smartphone behavior as being problematic, and a fourth (24%) often considered reducing their smartphone use (Table 1). Students used their smartphones for an average of 4 h and 54 min on weekdays and 5 h and 33 min on weekends (not shown in the table).

In the oldest age group (≥35 years), students exhibited a significantly lower tendency towards high risk of smartphone addiction compared to other age groups.

Students at high risk of smartphone addiction were found spending significantly more time with friends (>3 days per week) when compared to those in the low-risk group who predominantly spent 0–1 days per week with friends (Table 1).

Students with high risk of smartphone addiction perceived their behavior as being problematic to a significantly higher degree and more often considered reducing their smartphone use. Among those categorized as high risk of smartphone addiction, 74% were conscious of their problematic smartphone behavior. In contrast, only 24% of the low risk group perceived their smartphone behavior as problematic (Table 1). Finally, the high risk group also reported significantly (p = <0.001) greater average smartphone usage per day, with 6 h and 38 min per weekdays and 7 h and 14 min on weekend days. This amounts to a total of 47 h and 38 min per week. Low risk students spent a weekly average of 32 h and 52 min on their smartphones (4 h and 22 min/weekday and 5 h and 2 min/weekend-day) (not shown in the Table).

The characteristics of the participating students’ health behavior can be found in Table 2. More than half (66%) of the participants engaged in organized sports 0–1 times per week and over one-third (38%) performed physical activities apart from organized sports >3 times a week. The majority of our study population (86%) rated their health-related quality of life as good, 64% rated their mental health and well-being as normal, and 68% rated their self-esteem as normal (68%) (Table 2).

**Table 2:** Health behavior of participating students.

		All		High risk of smartphone addiction		Low risk of smartphone addiction		P-value
		*n*	%	*n*	%	*n*	%	
Overall *n*		1252-1295^a^		287		956		
**Participation in organized sport**								0.370
0–1 times/week		848	65.5	195	67.9	624	65.3	
2–3 times/week		292	22.5	65	22.6	213	22.3	
>3 times/week		155	12.0	27	9.4	119	12.4	
Mean ±(SD)		1.3 (±1.8)	–	1.2 (±1.6)	–	1.3 (±1.8)	–	
**Physical activity besides organized sport**								**0.030**
0–1 times/week		319	24.7	83	28.9	224	23.4	
2–3 times/week		487	37.7	115	40.1	358	37.4	
>3 times/week		486	37.6	89	31.0	374	39.1	*
Mean ±(SD)		3.1 (±2.1)	–	2.8 (±2.0)	–	3.2 (±2.1)	–	
**Health related quality of life**								**<0.001**
Good		1107	86.2	223	77.7	848	88.7	
Poor		177	13.8	64	22.3	108	11.3	
**Mental health and well-being (0**–**100)**								**<0.001**
Normal		809	63.8	142	49.5	651	68.1	
Low		459	36.2	145	50.5	305	31.9	
Mean ±(SD)		55.4 (±19.2)	–	48.7 (±19.0)	–	57.4 (±18.8)	**-**	
**Self-esteem (0-30)**								**<0.001**
Normal		850	67.9	164	57.1	680	71.1	
Low		402	32.1	123	42.9	276	28.9	
Mean ±(SD)		18.0 (±5.7)	–	16.4 (±5.3)	–	18.5 (±5.7)	–	

*: <0.05, **: <0.01 ***: <0.001.

^b^ = *behind the percentages indicates significant differences in rates between the respective sub-group and both other groups.

a Sample size for the different analyses diverges due to varying numbers of valid answers for the change assessments.

When taking into account high-risk and low-risk groups, statistically significant differences transpired. Fewer students with high risk of smartphone addiction reported engaging in physical activities other than organized sports >3 times per week. Concurrently, students with high risk of smartphone addiction also reported lower health-related quality of life, mental health and well-being, and self-esteem. No significant differences were detected concerning participating in organized sports. (Table 2).

### 3.2 Health behavior differences among students with high risk of smartphone addiction distributed on perceived behavior

The multivariate analysis exclusively included students with a high risk of smartphone addiction. The unadjusted analysis showed that students who self-identified their behavior as problematic were more than two times more likely to report poor (as compared to good) health-related quality of life (OR: 2.17, CI: 1.04–4.53) and low (as compared to normal) mental health and well-being (OR: 2.16, CI: 1.25–3.73) compared to those at high risk of smartphone addiction who were not identifying their behavior as problematic. However, in adjusted analyses, only associations with mental health and well-being remained significant (OR: 2.38, CI: 1.19–4.74) (see Table 3).

Model 1 Conducted separately for each independent variable. Raw analysis for each variable. Model 2: Fully adjusted. Conducted concurrently for all variables.

**Table 3:** Multivariate association between mental and physical health and perceived problematic behavior by students identified as being at high risk of smartphone addiction (*n* = 286).

	Perceived problematic behavior			
	Model 1		Model 2	
	OR	95% CI	OR	95% CI
**Gender**				
Men	1		1	
Women	1.44	0.73–2.84	1.67	0.80–3.47
**Age**				
≤24 years	1		1	
25–34 years	1.62	0.88–2.98	1.76	0.92–3.38
≥35 years	1.03	0.47–2.24	0.90	0.37–2.17
**Living with others**				
None	1		1	
1 person	1.11	0.57–2.14	1.21	0.60–2.46
≥2 persons	1.29	0.65–2.55	1.30	0.60–2.79
**Together with friends beside study**				
0–1 day/week	1		1	
2–3 days/week	0.93	0.52–1.67	1.22	0.62–2.40
>3 days/week	0.49	0.23–1.03	0.51	0.22–1.17
**Student job**				
No	1		1	
Yes	1.19	0.70–2.04	1.23	0.68–2.22
**Participation in organized sport**				
0–1 times/week	1		1	
2–3 times/week	0.97	0.51–1.87	1.11	0.54–2.28
>3 times/week	0.46	0.20–1.06	0.44	0.17–1.15
**Physical activity besides organized sport**				
0–1 times/week	1		1	
2–3 times/week	0.84	0.44–1.63	1.06	0.52–2.16
>3 times/week	0.76	0.38–1.52	1.38	0.62–3.06
**Health related quality of life**				
Good	1		1	
Poor	2.17*	**1.04**–**4.53**	2.01	0.85–4.79
**Mental health and well-being**				
Normal	1		1	
Low	2.16**	**1.25**–**3.73**	2.38*	**1.19**–**4.74**
**Self-esteem**				
Normal	1		1	
Low	0.98	0.57–1.67	0.53	0.27–1.05

*P < 0.05, **P < 0.01.

## 4 Discussion

The aim of this study was to investigate the potential risk of smartphone addiction and perceived problematic smartphone behavior among university college students. Results showed that 23% of the respondents were at high risk of smartphone addiction. Of these, the majority (74%) identified their smartphone behavior as problematic, with almost all expressing interest in reducing their smartphone usage.

Gender variations in smartphone addiction have been noted in previous cross-sectional studies, with women being considered more susceptible [9,19,31,32]. However, our findings did not show any significant gender differences, which may be due to the under-representation of men (18%) in our study sample. However, caution must be made when comparing the results of the existing research since the study by Celikkalp et al. [19] and Demirci et al. [9] did not take different gender cut-off-scores for SAS-SV into account but only investigated gender differences in mean SAS-SV score.

Consistent with existing cross-sectional studies [9,17], our research also found a lower prevalence rate of high-risk smartphone addiction amongst older students. An explanation for this age difference could be that the students in the older age group were introduced to smartphones later in life, which can have influenced their smartphone behavior, as an association between acquiring a smartphone at an early age and smartphone addiction has been shown in previous research [7]. Alternatively, interest diversion with advancing age, such as attention to family and children might explain this lower prevalence. Further investigation would be helpful to develop potential intervention strategies.

Our study revealed that students with a high risk of smartphone addiction tended to be less “often physically active besides organized sport”. This findings aligns with previous cross-sectional studies which suggest an association between smartphone addiction and reduced physical activity [18,33] and between problematic smartphone use and higher levels of sedentary behavior [18,34]. Another cross-sectional study found no significant differences between physical activity and the prevalence of smartphone addiction [31]. In the related area of organized sports, previous research has found an association between a high risk of smartphone addiction and a lower degree of participation in regular sports [19]. A finding not confirmed in our study, where no significant difference was observed regarding participating in organized sports amongst the high and low-risk groups. Diverse results across studies may stem from varying methods used for measuring physical activity levels and involvement in sports.

Additionally, our results indicate a higher prevalence of poor health-related quality of life, compromised mental health and well-being, and lower self-esteem amongst the high-risk smartphone users. This is consistent with a recent systematic review of smartphone addiction and associated health outcomes in adult populations [35], which included 27 cross-sectional studies. Their findings suggest a consistent association between smartphone addiction and poor mental health. Other recent studies hint at a non-linear association between screen time [36] or time spent using smartphones [37] and mental health and well-being. Their results support the Goldilock hypothesis that suggests that moderate use of digital technology is not intrinsically harmful and may instead be beneficial, whereas none or excessive use seems to be associated with poorer mental health [51].

A recent cross-sectional study observed a significant negative effect of “unaware use” of smartphones on quality of life, but reported a mild positive effect when using in an “aware mode” [52]. Others have explained the association between smartphone addiction and low levels of mental health with a tendency to check notifications all the time (reassurance seeking) in those individuals whose smartphone use is driven by the necessity to maintain relationships and obtain reassurance from others [35,53]. This suggests that the association we found between high risk of smartphone addiction and poor health-related quality of life and low levels of mental health and well-being might be influenced by the style of smartphone usage, warranting further exploration.

To our knowledge, this is one of the first studies on smartphone addiction that also investigated whether young people perceived their smartphone usage as problematic. A majority (74%) of students with a high risk of smartphone addiction did identify their smartphone behavior as being problematic, and many (40%) often considered reducing their smartphone use. As expected, distinctively fewer among the low-risk group did so (24% perceived their behavior as problematic and 19% often considered reducing their smartphone use).

The fact that 74% of students with a high risk of smartphone addiction acknowledge their behavior as problematic might indicate a readiness to modify their behavior eventually [29]. Health educators and policymakers could use students’ perceptions of their behavior as a starting point to identify barriers and strategies with the students.

We found no significant differences between students with a high risk of smartphone addiction who perceived their smartphone behavior as problematic (as compared to high risk but not problematic) on sociodemographic characteristics, physical health, health-related quality of life, and self-esteem. However, they significantly more often reported low mental health and well-being. To our knowledge, young people's perception of their smartphone behavior has not been investigated in a high-risk group before. It might be important knowledge in future considerations regarding behavior interventions targeting students with high risk of smartphone addiction.

### 4.1 Strengths and limitations

In interpreting the results of our study, it's crucial to acknowledge its limitations. Firstly, our study utilizes a cross-sectional survey design, which enables us to identify relationships, but not causal links, between smartphone addiction risks, sociodemographic characteristics, physical health, and mental health. Second, the relatively low response rate (a common issue with online surveys [54]), and significant differences in age and gender distribution compared to the eligible study population may limit the study's representativeness and generalizability. Therefore, it is imperative to exercise caution when applying these findings more broadly. The percentage of students with a high risk of smartphone addiction might be slightly overestimated due the higher percentage of women who responded, but on the other hand, it might have slightly underestimated the percentage, due to the significant higher response rate among students aged ≥35. Thirdly, the study relies upon a convenience sample of young Danish students. So, extrapolating these results beyond this particular demographic (≥18 years old university college students studying health science, education, and social administration) requires care. However, sampling among university and college students is very common in this field.

Fourthly, we adapted the cut-off score on the SAS-SV, validated on a Korean population [23], due to the absence of a validated Danish cut-off score. Although researchers worldwide have used the same cut-off score [17,31,55],. an investigation discovered heterogeneous cut-off scores in different Spanish-speaking populations using standard procedures (i.e. reliability, validity, ROC methodology) [56]. This findings suggests caution is necessary when using a SAS-SV cut-off score not validated explicitly for your study population. However, among the numerous scales to assess smartphone addiction and problematic smartphone use, a recent systematic review [28] which included 78 validated scales, concluded that based on internal consistency, the SAS-SV by Kwon et al. [23] was among the three most reliable scales. We are aware of the reduction of data when we dichotomize the scale by established cut-off points. The SAS-SV scale could have been used in the analysis as a continuous scale. Still, as our aim using the SAS-SV was to identify those at high risk of addiction, we chose to use the proposed cut-off [23] to be able to compare results with other studies looking at the risk of smartphone addiction.

Finally, fundamentally in using surveys, the aspect studied is assessed subjectively. The use of self-reporting in surveys may lead to overestimation or underestimation, introducing potential bias. While we used validated and well-known scales wherever possible, some variables were developed and pilot tested by our research team, which further adds an element of subjectivity. To our knowledge, no tool was developed to measure “self-assessment of perceived problematic smartphone use” and “smartphone reduction considerations”, as this study is the first to investigate this in correlation with smartphone addiction. Thus, it is unknown if the questions asked fully measure these variables, but face validity and feasibility was investigated. Further, there was a risk of type I error when dichotomizing these variables.

Strengths of our study include the examination of a Nordic sample, adding diversity to the predominantly Asian-based studies in the field. Additionally, this study featured a large number of participants, enhancing its statistical power. Lastly, the multivariate analysis results remain consistent when using continuous variables, indicating a low risk of false positives due to dichotomizing variables. This suggests our findings are robust and reliable.

## 5 Conclusion

Conclusively, this study found that one in four students were at high risk of smartphone addiction. A significant segment, three-fourths, within this high-risk group perceived their smartphone behavior as problematic, with the majority contemplating reducing their smartphone use. Thus, these findings suggest a desire amongst these students to modify their excessive smartphone habits. Additionally, students who recognized their high-risk smartphone usage as problematic were more likely to report low mental health and well-being scores.

These correlations brings to light valuable insights for creating practical strategies as it suggests students at high-risk of smartphone addiction—those who consider their usage problematic—would be receptive to support and behavioral change initiatives. Considering the association of low mental health and well-being in this group, the results highlight the need for future scientific research and applied focus on engaging heavy smartphone users in co-developing personalized interventional strategies. Such collaborative efforts might pave the way for a balanced digital lifestyle and improved mental health.

## Declaration

During the preparation of this work, the authors used the AI tool “Jenni” in order to improve language and readability. After using this tool, the authors reviewed and edited the content as needed and takes full responsibility for the content of the publication.

## Funding

This research was funded by The 10.13039/100010324Research center at University College South (UCSYD), Denmark.

## Declaration of competing interest

The authors declare that they have no known competing financial interests or personal relationships that could have appeared to influence the work reported in this paper.

## Footnotes

Appendix A Supplementary data to this article can be found online at https://doi.org/10.1016/j.heliyon.2023.e20368.
