# Supplementary material for: Do young people perceive their smartphone addiction as problematic? A study in Danish university college students
Source: Heliyon. 2023 Sep 25;9(10):e20368. doi: 10.1016/j.heliyon.2023.e20368 (PMC10543362; doi:10.1016/j.heliyon.2023.e20368)
Supplement: Multimedia component 1 [file mmc1.pdf]

Appendix 1. Applied questions in the survey of Problematic Smartphone use.

Variable	Question	Response Categories
	**Thank you for participating in the survey.**<br><br>The questionnaire is about your smartphone use, your health, activities, etc.<br><br>Your participation is voluntary and answers anonymous.	
	**The first questions are about your smartphone usage.**	
Smartphone ownership	Do you own a smartphone?	Yes/No
Considerations for reducing smartphone use	How often do you consider reducing your smartphone use?	Never or almost never/ Sometimes/ Most of the time/ All the time or almost all the time
Self-assessment of perceived problematic smartphone use	To which degree do you agree with the following statement? I consider my use of my smartphone as problematic	Strongly disagree/ Disagree/ Slightly disagree/ Slightly agree/ Agree/ Strongly agree
Risk of smartphone addiction: SAS-SV	Below are ten questions about your smartphone use. (Please tick one for each statement<br>1. Missing planned work due to smartphone use<br>2. Having a hard time concentrating in class, while doing assignments, or while working due to smartphone use<br>3. Feeling pain in the wrists or at the back of the neck while using a smartphone<br>4. Won't be able to stand not having a smartphone<br>5. Feeling impatient and fretful when I am not holding my smartphone<br>6. Having my smartphone in my mind even when I am not using it<br>7. I will never give up using my smartphone even when my daily life is already greatly affected by it<br>8. Constantly checking my smartphone so as not to miss conversations between other people on Twitter or Facebook<br>9. Using my smartphone longer than I had intended<br>10. The people around me tell me that I use my smartphone too much	Strongly disagree/ Disagree/ Weakly disagree/ Weakly agree/ Agree/ Strongly agree

Amount of smartphone use	Within the past four weeks, how much time have you typically spent per WEEKDAY on your smartphone on the following screen-based activities?<br><br>Please tick one per screen activity per WEEKDAY (You will be asked for the weekend day in the next question).<br><br>Please tick the category ”none”, if there is a screen activity you do not use time on.<br><br>1. Watching TV (eg movies, TV shows, TV series, entertainment programs): Weekday<br>2. Playing games: Weekday<br>3. Talking on the phone (eg via mobile phone, Face Time or Skype): Weekday<br>4. Social media or ways to communicate with others (eg Facebook, Twitter, Instagram, Snapchat, SMS og E-mail): Weekday<br>5. Surfing the web (such as reading, shopping, going on YouTube): Weekday<br>6. Other things (eg taking pictures, viewing or editing pictures, etc.): Weekday	None/ 1-29 minutes/ 30-59 minutes/ 1-2 hours/ 3-4 hours/ 5 hours or more
Amount of smartphone use	Within the past four weeks, how much time have you typically spent per WEEKEND DAY on your smartphone on the following screen-based activities?<br><br>Please tick one per screen activity per WEEKEND DAY.<br><br>Please tick the category ”none”, if there is a screen activity you do not use time on.<br><br>1. Watching TV (eg movies, TV shows, TV series, entertainment programs): Weekend day<br>2. Playing games: Weekend day<br>3. Talking on the phone (eg via mobile phone, Face Time or Skype): Weekend day	None/ 1-29 minutes/ 30-59 minutes/ 1-2 hours/ 3-4 hours/ 5 hours or more

	4. Social media or ways to communicate with others (eg Facebook, Twitter, Instagram, Snapchat, SMS og E-mail): Weekend day<br>5. Surfing the web (such as reading, shopping, going on YouTube): Weekend day<br>6. Other things (eg taking pictures, viewing or editing pictures, etc.): Weekend day	
	**The second section contains questions about your everyday life**	
Sociodemographic variables – together with friends	How often are you together /out with friends outside study time?	0/ 1/ 2/ 3/ 4/ 5/ 6/ 7 days/week
Sociodemographic variables – students job	Do you have a student job alongside your studies?	Yes/ No
Sociodemographic variables – student job, hours	If yes, how many hours do you work per week?	Hours:_____
Physical activity – organized sport	How often do you participate in organized sport during a week (sports club, fitness, outdoor, dance, etc.)?	0/ 1/ 2/ 3/ 4/ 5/ 6/ 7 or more times/week
Physical activity- beside organized sport	How often are you physically active besides organized sports (bike rides, runs, walks, gardening, or similar activities)?	0/ 1/ 2/ 3/ 4/ 5/ 6/ 7 or more times/week
	**The third section contains questions about your health**	
Health-related quality of life: from SF36	In general, would you say your health is:	Excellent/ Very good/ Good/ Fair/ Poor
Mental health and well-being: WHO5	Please indicate for each of the five statements, which is closest to how you have been feeling in the past two weeks.<br>1. … I have felt cheerful and in good spirits<br>2. … I have felt calm and relaxed<br>3. … I have felt active and vigorous<br>4. … I woke up feeling fresh and rested<br>5. … My daily life has been filled with things that interest me	At no time/ Some of the time/ Less than of the time/ More than half of the time/ Most of the time/ All the time

Self-esteem: Rosenberg Self Esteem Scale	Please indicate how strongly you agree or disagree with each of the following statements<br>1. On the whole, I am satisfied with myself.<br>2. At times I think I am no good at all.<br>3. I feel that I have a number of good qualities.<br>4. I am able to do things as well as most other people.<br>5. I feel I do not have much to be proud of.<br>6. I certainly feel useless at times.<br>7. I feel that I'm a person of worth, at least on an equal plane with others.<br>8. I wish I could have more respect for myself.<br>9. All in all, I am inclined to feel that I am a failure.<br>10. I take a positive attitude toward myself.	Strongly disagree/ Disagree/ Agree/ Strongly agree
	**The last section contains some general questions about you**	
Sociodemographic variables – Gender	How do you identify as gender?	Man/ Women/ Other
Sociodemographic variables – Age	How old are you?	< 20 years/ 20 – 24 / 25 – 29 / 30 – 34 / 35 – 39 / 40 years or more
Sociodemographic variables – Study program	Which education/study program are you currently following?	Dietitian/ English and digital marketing communication/ Graphic communication/ Health administrative coordinator / Kindergarten teacher / Laboratory technician/ Medical laboratorian technologist/ Midtwife/ Nurse/ Occupational therapist / Pedagogical assistant/ Physiotherapist/ Schoolteacher/ Social worker/ Sound design/ Tax and public administration
Sociodemographic variables- number of roommates	How many people do you live with, besides yourself?	0/ 1/ 2/ 3/ 4/ 5/ 6/ 7 or more
