# Supplementary material for: Do young people perceive their smartphone addiction as problematic? A study in Danish university college students
Source: Heliyon. 2023 Sep 25;9(10):e20368. doi: 10.1016/j.heliyon.2023.e20368 (PMC10543362; doi:10.1016/j.heliyon.2023.e20368)
Supplement: Multimedia component 2 [file mmc2.pdf]

Appendix 2. The Danish version of applied questions in the survey of Problematic Smartphone use. (Anvendte spørgsmål i undersøgelsen om Problematisk Smartphone Use).

Variable – in Danish	Question – in Danish	Response Categories – in Danish
	**Tak fordi du vil deltage i undersøgelsen.**<br><br>Spørgeskemaet handler om dit smartphoneforbrug, dit helbred m.m.<br><br>Din deltagelse er frivillig, og besvarelsen er anonym.	
	**De første spørgsmål handler om dit smartphoneforbrug**.	
Smartphone ejerskab	Har du en smartphone?	Ja/Nej
Overvejelser i forhold til at nedsætte smartphone forbrug	Hvor ofte overvejer du at nedsætte dit smartphoneforbrug?	På intet eller næsten intet tidspunkt/ Nogle gange/ For det meste/ Hele tiden eller næsten hele tiden
Selvvurdering af om ens smartphoneforbrug er problematisk	Hvor enig er du I følgende udsagn? Jeg har et problematisk forbrug af smartphone	Meget uenig/ Uenig/ Lidt uenig/ Lidt enig/ Enig/ Meget enig
Risiko for smartphone afhængighed: SAS-SV	Herunder kommer ti spørgsmål omkring dit smartphoneforbrug. (Angiv ét kryds for hvert udsagn)<br>1. Der er planlagt arbejde, som jeg ikke får udført, fordi jeg bruger tid på min smartphone<br>2. Jeg har svært ved at koncentrere mig, når jeg har undervisning, laver hjemmeopgaver eller når jeg er på arbejde, fordi jeg bruger min smartphone<br>3. Jeg oplever smerte i håndled eller nakke, når jeg bruger smartphone<br>4. Jeg vil ikke kunne holde ud at undvære en smartphone<br>5. Jeg bliver utålmodig og gnaven, hvis jeg ikke har min smartphone i hånden<br>6. Jeg har min smartphone i tankerne, selv når jeg ikke bruger den<br>7. Selv hvis det I høj grad påvirkede min hverdag, ville det ikke afholde mig fra at bruge min smartphone<br>8. Jeg tjekker konstant sociale medier (fx Twitter, Facebook) på min smartphone, så jeg ikke går glip af andre personers samtaler	Meget uenig/ Uenig/ Lidt uenig/ Lidt enig/ Enig/ Meget enig

	9. Jeg bruger min smartphone i længere tid end jeg har til hensigt at bruge den<br>10. Min omgangskreds gør mig opmærksom på, at jeg bruge min smartphone for meget	
Mængde af smartphoneforbrug	Hvor meget tid har du gennemsnitligt brugt om dagen på din smartphone indenfor de sidste 4 uger i HVERDAGEN?<br><br>Sæt venligst ét kryds pr. skærm-aktivitet per HVERDAG (du bliver spurgt til weekenddag i næste spørgsmål).<br><br>Sæt venligst kryds i kategorien ”ingen”, hvis der er skærm aktivitet, som du ikke bruger tid på.<br>1. Se fjernsyn (f.eks. film, TV-serie, underholdningsprogrammer): Hverdag<br>2. Spille spil: Hverdag<br>3. Telefonsamtale (f.eks. via mobiltelefon, FaceTime eller skype): Hverdag<br>4. Sociale medier eller måder at skrive sammen med andre på (f.eks. Facebook, Twitter, Instagram, Snapchat, SMS og E-mail): Hverdag<br>5. Surfe på nettet (såsom at læse nyheder, handle, gå på YouTube) : Hverdag<br>6. Andet (f.eks. tage billeder, se eller redigere billeder mm.): Hverdag	Ingen/ 1-29 minutter/ 30-59 minutter/ 1-2 timer/ 3-4 timer/ 5 timer eller mere
Mængde af smartphoneforbrug	Hvor meget tid har du gennemsnitligt brugt om dagen på din smartphone indenfor de sidste 4 uger i WEEKENDEN?<br><br>Sæt venligst ét kryds pr. skærm-aktivitet per weekenddag.<br><br>Sæt venligst kryds i kategorien ”ingen”, hvis der er skærm aktivitet, som du ikke bruger tid på.<br>1. Se fjernsyn (f.eks. film, TV-serie, underholdningsprogrammer): Weekend dag<br>2. Spille spil: Weekend dag<br>3. Telefonsamtale (f.eks. via mobiltelefon, FaceTime eller skype): Weekend dag	Ingen/ 1-29 minutter/ 30-59 minutter/ 1-2 timer/ 3-4 timer/ 5 timer eller mere

	4. Sociale medier eller måder at skrive sammen med andre på (f.eks. Facebook, Twitter, Instagram, Snapchat, SMS og E-mail): Weekend dag<br>5. Surfe på nettet (såsom at læse nyheder, handle, gå på YouTube) : Weekend dag<br>6. Andet (f.eks. tage billeder, se eller redigere billeder mm.): Weekend dag	
	**Det andet afsnit indeholder spørgsmål omkring din hverdag**	
Sociodemografisk variabel – sammen med venner	Hvor mange eftermiddage/aftener om ugen er du normalt ude/sammen med venner (ud over studietid)?	0/ 1/ 2/ 3/ 4/ 5/ 6/ 7 dage/uge
Sociodemografisk variabel – studie job	Har du arbejde ved siden af dit studie?	Ja/ Nej
Sociodemografisk variabel – studie job, i timer	Hvis ja, hvor mange timer om ugen arbejder du?	Timer:_____
Fysisk aktivitet – organiseret sport	Hvor mange gange om ugen går du til organiseret sport (klub sport, fitness, outdoor, dans osv.)	0/ 1/ 2/ 3/ 4/ 5/ 6/ 7 eller flere gange/uge
Fysisk aktivitet- udover organiseret sport	Hvor mange gange om ugen dyrker du sport eller er aktiv på egen hånd (cykelture, løbeture, gåture, havearbejde eller lign)	0/ 1/ 2/ 3/ 4/ 5/ 6/ 7 eller flere gange/ uge
	**Det tredje afsnit indeholder spørgsmål omkring dit helbred**	
Selvvurderet helbred/livskvalitet: Fra SF36	Hvordan synes du, dit helbred er alt ialt:	Fremragende/ Vældig godt/ Godt/ Mindre godt/ Dårligt
Mental sundhed og well-being: WHO5	Sæt venligst, ved hvert af de fem udsagn, et kryds i det felt der kommer tættest på, hvordan du har følt dig i de seneste to uger.<br>1. … Jeg har været glad og i godt humør<br>2. … Jeg har følt mig rolig og afslappet<br>3. … Jeg har følt mig aktiv og energisk<br>4. … Jeg er vågnet frisk og udhvilet<br>5. … Min dagligdag har været fyldt med ting der interesserer mig	På intet tidspunkt/ Lidt af tiden/ Lidt mindre end halvdelen af tiden/ Lidt mere end halvdelen af tiden/ Det meste af tiden/ Hele tiden
Selvværd: Rosenberg Self Esteem Scale	Angiv venligst hvor enig du er i følgende udsagn.<br>1. Du er altid tilfreds med dig selv.<br>2. Fra tid til anden synes du, at du overhovedet ikke dur til noget.	Stærkt uenig/ Uenig/ Enig/ Meget enig

	3. Du synes, at du har en del gode egenskaber.<br>4. Du er i stand til at gøre noget lige så godt, som de fleste andre.<br>5. Du synes, at du ikke har meget at være stolt af.<br>6. Du føler dig helt nytte løs fra tid til anden.<br>7. Du synes, at du er et menneske af værdi. I det mindste lige så værdifuld som de fleste andre.<br>8. Du ville ønske, at du kunne have mere respekt for dig selv.<br>9. Alt I alt er du tilbøjelig til at tro, at du er mislykket.<br>10. Du har en positive holdning over for dig selv.	
	**Det sidste afsnit indeholder spørgsmål omkring dig generelt**	
Sociodemografisk variabel – Køn	Hvad er dit køn?	Mand/ Kvinde/ Andet
Sociodemografisk variabel – Alder	Hvor gammel er du?	< 20 år/ 20 – 24 / 25 – 29 / 30 – 34 / 35 – 39 / 40 år eller derover
Sociodemografisk variabel – Uddannelsesretning	Hvilken studieretning går du på?	Bioanalytiker/ Engelsk og digital markedskommunikation/ Ergoterapeut/ Ernæring og sundhed/ Fysioterapeut/ Grafisk kommunikation/ Jordemoder/ Laborant/ Lyddesign (sono)/ Lærer/ Pædagog/ Pædagogisk assistent/ Skat eller administrationsbachelor/ Socialrådgiver/ Sundhedsadministrativ koordinator/ Sygeplejerske
Sociodemografisk variabel- Husstand størrelse	Hvor mange bor der i din husstand udover dig selv?	0/ 1/ 2/ 3/ 4/ 5/ 6/ 7 eller flere
